# Formation of Monocyclic and Polycyclic Hydrocarbons from Sequential Toluene Reactions in Coulomb Crystals

**DOI:** 10.1021/acs.jpca.6c01167

**Published:** 2026-05-19

**Authors:** G. S. Kocheril, C. Zagorec-Marks, S. H. Allison, H. J. Lewandowski

**Affiliations:** † JILA, National Institute of Standards and Technology, and 172322 the University of Colorado, Boulder, Colorado 80309, United States of America; ‡ Department of Physics, 1877 University of Colorado, Boulder, Colorado 80309, United States of America; § Department of Chemistry, University of Colorado, Boulder, Colorado 80309, United States of America

## Abstract

Understanding the formation and reactivity of aromatic molecules is important for understanding chemistry in a wide range of environments, including the interstellar medium. The clear observation of polycyclic aromatic hydrocarbons (PAHs) in space has intensified efforts to identify efficient formation pathways capable of producing such complex species under cold, dilute conditions. In astrochemical environments, ion–molecule reactions are typically the dominant type of reactions due to their high efficiency compared to neutral–neutral reactions. However, current models of PAH formation largely neglect ion–molecule reactions beyond the initial formation of benzene. Here, we present results from a series of ion–molecule reactions carried out in Coulomb Crystals beginning with the protonation of toluene to identify possible reaction pathways that could lead to interstellar PAH growth. In this work, we observe the formation of monocyclic aromatic species, C_6_H_5_^+^ and C_7_H_7_^+^, via the initial dissociative proton transfer to toluene. We also observe an associative proton transfer product, resulting in C_7_H_9_^+^. We find the sequential reaction between C_7_H_9_^+^ and C_7_H_8_ results in the formation of C_7_H_5_^+^, C_12_H_5_^+^, and C_12_H_8_^+^, which provides the first experimental evidence of a possible interstellar formation mechanisms of polycyclic cations beginning with monocyclic precursors.

## Graphical abstract

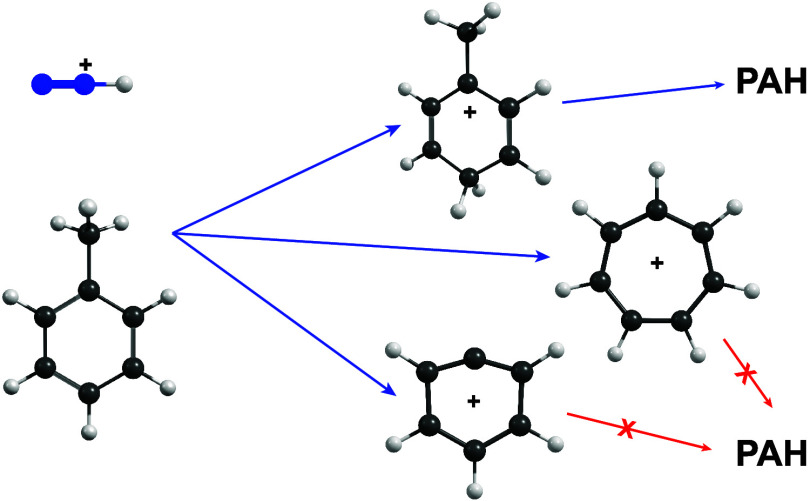

## Introduction

Aromatic molecules have captivated chemists since Faraday’s discovery of benzene over 200 years ago.^1^ Although the specifics of the structure, bonding, and even chemical formula were not known at this time, the minimal reactivity of the isolated compound alerted the chemists of the time to a new molecular property. Significant work has been carried out over the past two centuries to better understand the intrinsic properties that govern this reactivity, and we now know that the structure of aromatic hydrocarbons are cyclical in structure and feature strong electron delocalization across the carbon framework.^2^ Aromatic molecules are found both industrially and in nature, and are known to add structural rigidity and chemical stability. In biochemistry, aromatic moieties are found in a few amino acids and nucleic acids, which stabilize the structures of proteins and DNA respectively.^3,4^ These same aromatic moieties are commonly found in pharmaceuticals and dyes due to their stabilizing effects.^5,6^ Aromatic molecules are also being considered as potential organic semiconductors because of their electronic properties.^7^ Observations of aromatic molecules have also expanded beyond hydrocarbons to other parts of the periodic table, as well as new forms of aromaticity that go beyond Hückel’s electron counting rule.^8,9^

Understanding the formation mechanisms of aromatic molecules in the gas-phase has gained heightened interest due to recent observations of polycyclic aromatic hydrocarbons (PAHs) in the interstellar medium (ISM).^10–18^ However, unlike in the condensed phase, there is a significant lack of understanding of how such aromatic molecules are formed in the extreme environments of the ISM.^15,19^ Interstellar PAHs are predicted to be produced by two main types of formation mechanisms, either a top-down or a bottom-up process.^20^ In the top-down mechanism, large macroscopic particles or grains are fragmented by harsh ultraviolet radiation into smaller components, ultimately yielding PAHs. For the bottom-up mechanism, small hydrocarbon molecules react and aggregate to eventually form PAH molecules. Ion–molecule reactions are often invoked to model the bottom-up formation of benzene, which is considered the rate-limiting step in the interstellar PAH growth process.^21–23^ Despite the relevance of ion–molecule reactions to benzene formation, such reactions are not included in models of chemical growth beyond benzene; instead, current models rely exclusively on neutral–radical reactions to predict the formation of observed PAHs.^19,24^ Generally, ion–molecule reaction rate constants are significantly larger than their neutral counterparts, yet this class of reactions has largely been excluded from PAH growth models due to a lack of experimental data demonstrating potential formation mechanisms.^15^

A valuable model system for exploring ion–molecule-reaction growth pathways to PAHs, even though it has not yet been observed in the interstellar medium, is protonated toluene (C_7_H_9_^+^), known as toluenium, which is the simplest alkylbenzenium ion. Studying the reactivity of toluenium enables insight into the reactivity of substituted benzene cations and their potential role in PAH formation throughout the ISM. Additionally, toluenium ions have been observed to undergo unimolecular dissociation immediately upon formation depending on the environment, motivating extensive interest in their formation mechanisms and reactivity.^25^ Prior studies have revealed a rich energy landscape, which allows for isomerization between various tautomers of toluenium, as well as other isomeric forms of C_7_H_9_^+^ under standard experimental conditions.^25–27^ This chemical complexity has motivated many experimental efforts to identify and characterize the various forms of toluenium.

As the most stable of all possible structural isomers of C_7_H_9_^+^, the structure of toluenium has been the subject of significant interest over the past several decades.^25^ In solution, it was shown that protonated toluene can take the form of four distinct tautomers, shown in Figure 1. At the ωB97X-D/aug-cc-pVTZ level of theory, we find that *para*-C_7_H_9_^+^ is the global minimum structure, with *ortho*-C_7_H_9_^+^ being 0.06 eV higher in energy. The other two tautomers are higher in energy, with the *meta*-C_7_H_9_^+^ lying 0.23 eV above the global minimum and the *ipso*-C_7_H_9_^+^ being the highest-energy structure at 0.42 eV above the global minimum. In super acidic solutions, *p*-C_7_H_9_^+^ was initially observed to be the global minimum by NMR,^28^ but it was also found that both the *o*- and *p*-tautomers could coexist in a super acid media due to the small energy difference between the two.^29^ The *p*-toluenium was later isolated by X-ray crystallography of toluenium-carborane salts, confirming it as the global minimum.^30^ Although tautomerically pure toluenium ions have been produced in the condensed phase, this has yet to be achieved in the gas phase. This inability to isolate tautomerically pure toluenium is largely attributed to a fast proton ring walk, which cannot be easily suppressed in the gas-phase due to negligible barriers.^25,27,31^

**Figure 1:**
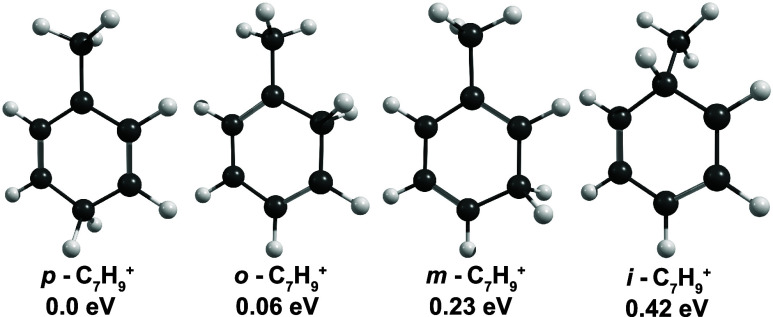
Four tautomers of toluenium, with zero-point energy corrected energies shown relative to *p*-C_7_H_9_^+^, calculated at the ωB97X-D/aug-cc-pVTZ level of theory.

Toluenium is a unique model system also because of the presence of multiple relatively low-energy unimolecular dissociation channels that are readily accessible upon formation via protonation by proton donors with various levels of acidity.^32^ This unimolecular fragmentation results in two highly stable monocyclic aromatic cations, namely tropylium (C_7_H_7_^+^), shown in eq 1 and phenylium (C_6_H_5_^+^), shown in eq 2. These dissociation channels have also been the subject of several studies where changes in product branching is used to indirectly probe the structure of the initial C_7_H_9_^+^ complex and elucidate the reaction mechanism along the reaction surface.^25^ Both tropylium and phenylium are of interest as they could play a role in the formation of interstellar PAHs. However, their reactivity is not well understood, so their prevalence in models is limited. Tropylium and phenylium production from dissociative protonation of toluene are less exothermic than the formation of toluenium, which is a direct associative protonation reaction (eq 3 where the exothermicity of this reaction is calculated assuming formation of the global minimum). This makes the choice of proton donor crucial to guarantee observation of all protonation channels of toluene.^32^ N_2_H^+^ is an ideal proton donor to probe both the associative and dissociative protonation channels of toluenium. Weaker acids, such as H_3_O^+^, are unable to input enough energy into the system to produce the fragmentation channels. In contrast, stronger acids, such as H_3_^+^, are expected to impart substantially more energy to the system, leading to extensive fragmentation and reducing the observable population of C_7_H_9_^+^.^32^ Using N_2_H^+^, therefore, enables access to both channels, while preserving enough stable C_7_H_9_^+^ to characterize its subsequent reactivity.

In the work presented here, we explore all product channels resulting from the N_2_H^+^ + C_7_H_8_ reaction, observing both associative and dissociative proton transfer products. We also characterize the products of secondary reactions between the three primary products and neutral toluene, which have not been studied previously. These sequential reactions reveal potential formation of polycyclic hydrocarbons, which introduces a new class of ion–molecule reaction sequences that can serve as possible formation routes to interstellar PAHs.

1 $$N_{2}H^{+}+C_{7}H_{8}\to C_{7}H_{7}^{+}+H_{2}+N_{2},\Delta E=-2.31\;\mathrm{eV}$$

2 $$N_{2}H^{+}+C_{7}H_{8}\to C_{6}H_{5}^{+}+{\mathrm{CH}}_{4}+N_{2},\Delta E=-0.46\;\mathrm{eV}$$

3 $$N_{2}H^{+}+C_{7}H_{8}\to C_{7}H_{9}^{+}+N_{2},\;\Delta E=-3.22\;eV$$

## Experimental Methods

The ion trapping apparatus used in this study has been described in detail previously,^33^ but a brief description of the apparatus is included here for clarity. The core of the apparatus is a linear, quadrupolar Paul trap, which confines all reactant and product ions. Initially, ∼900 Ca^+^ ions are loaded into the trap via nonresonant photoionization (Continuum Minilite Nd:YAG, ∼7 mJ/pulse, 355 nm, 10 Hz) of an effusive beam of Ca from an oven source. The trapped Ca^+^ ions are laser cooled to sub-Kelvin temperatures (∼100 mK) using the doubled output of a Ti-Sapphire laser (MSquared SolsTiS, ∼397 nm, 2 mW) and a diode laser (Toptica DL Pro, ∼866 nm, 2 mW). As the Ca^+^ are laser-cooled inside the trap, the ions form a pseudocrystalline structure known as a Coulomb crystal. The Coulomb crystal was used to sympathetically cool all molecular ions formed in this study to translational temperatures on the order of 1 K. The ion trap is housed in an ultrahigh vacuum (UHV) chamber, which has a background pressure of ∼3 × 10 ^– 10^ Torr. This pressure is measured using a Bayard-Alpert ionization gauge, which has reduced accuracy at pressures below 10^–8^ Torr.^34^ As a result, the uncertainties provided represent statistical uncertainties in the measurements and do not reflect the accuracy of the ionization gauge.

Once the Ca^+^ Coulomb crystal is formed, N_2_H^+^ ions (∼90 ions) are generated by first loading N_2_^+^ ions into the trap through a 2 + 1 Resonance Enhanced Multi-Photon Ionization (REMPI) scheme^35^ using a frequency doubled pulsed-dye laser (LIOPTEC Liopstar, 237 nm, ∼0.7 mJ/pulse, 10 Hz). Once trapped, the N_2_^+^ ions were allowed to react with trace residual water and molecular hydrogen in the vacuum chamber over the course of 2 min to form the desired N_2_H^+^ ions. After the ∼90 N_2_H^+^ were produced, reactions are initiated by introducing gas-phase neutral toluene (Thermo Fischer Scientific, 99.5% purity) 6% seeded in He into the vacuum system through a pulsed leak valve. The pressure of the neutral reactant is low enough (1.2 × 10^–9^ Torr partial pressure) that single collision conditions are assured. As a reaction proceeds, all ionic products are retained in the trap and analyzed at various reaction time points by turning off the trap and ejecting all trapped ions into a time-of-flight mass spectrometer (TOF-MS). By monitoring the number of reactant and product ions as a function of reaction time (0–350 s), we are able to construct kinetic reaction curves.

## Theoretical Methods

Potential energy surfaces were explored for the N_2_H^+^ + C_7_H_8_ reaction via quantum chemical calculations performed using Gaussian 16 at the unrestricted ωB97X-D/aug-cc-pVTZ level of theory to identify plausible barrierless formation routes.^36^ Each surface was computed starting with the reactants at infinite separation, with stationary points along the surface identified until the system reached the final observed product. Scans over bond lengths, angles, and dihedrals were used for identifying minima and potential transition states. These potential maxima were explored using optimization and frequency calculations. Transition states were identified by the presence of a vibrational mode with an imaginary frequency and through use of intrinsic reaction coordinate calculations. Only barrierless pathways were considered because of the energetic constraints imposed by the low temperatures of the reactions.

## Results

### Primary Products

We observe three unique products as the result of the N_2_H^+^ + C_7_H_8_ reaction, with mass-to-charge ratios (*m*/*z*) of 77, 91, and 93. As we are working in an UHV system with very limited background gas contaminants, each mass can be readily assigned as a pure hydrocarbon ion. As such, we assign *m*/*z* = 77 as C_6_H_5_^+^, *m*/*z* = 91 as C_7_H_7_^+^, and *m*/*z* = 93 as C_7_H_9_^+^. The kinetic reaction curves of the initial proton transfer reaction are shown in Figure 2, indicating the formation of three primary products. Each experimental data point corresponds to seven measurements that were normalized to the fitted initial number of N_2_H^+^ ions. The curves represent a global fit to all data (ionic reactant, primary and secondary product channels) using a pseudo-first-order rate law. The observed reactions are summarized in eqs 1, 2, and 3 for clarity. In these reactions, the least abundant reaction product is C_6_H_5_^+^, which accounts for ∼7% of the total products and is observed to grow slowly and plateau over time. This plateauing signal indicates that C_6_H_5_^+^ is a terminal product, which is unreactive with neutral toluene. The most abundant product is C_7_H_7_^+^, which is observed to be ∼60% of the total products. After an initial rise at the early time points, C_7_H_7_^+^ begins to plateau, which again indicates that it is a terminal product and does not react further with neutral toluene. The second most abundant product is C_7_H_9_^+^, which at its peak, accounts for ∼20% of the total products. At long reaction times (≥100 s), the C_7_H_9_^+^ signal begins to deplete, indicating further sequential reactions with neutral toluene leading to the formation of new products, shown in Figure 3.

**Figure 2:**
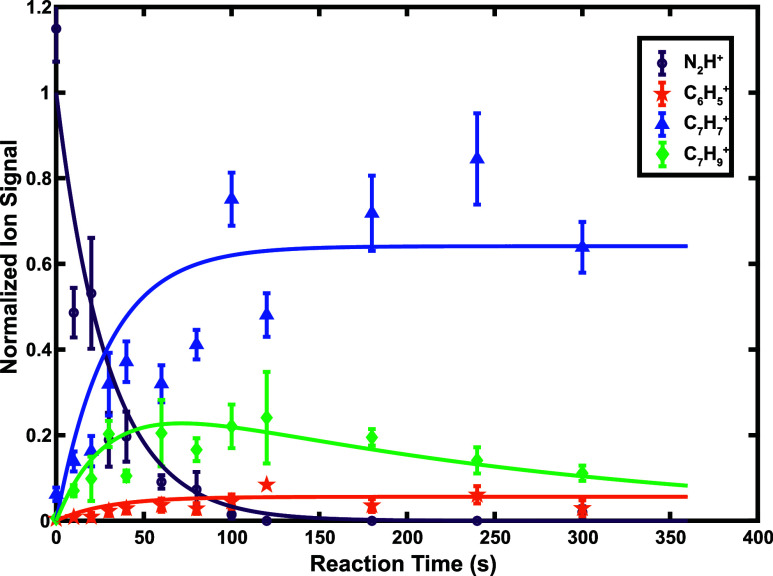
Kinetic reaction curves for the observed primary products for the proton transfer reaction N_2_H^+^ + C_7_H_8_. Experimental data points were fit using a pseudo-first order rate law (curves). Each data point corresponds to seven measurements that were normalized to the fitted initial number of ∼90 N_2_H^+^ ions. The purple points correspond to the N_2_H^+^ ions, which exhibit a sharp decay as they react with neutral toluene. This reaction produces three products, C_7_H_9_^+^ (green diamonds), C_7_H_7_^+^ (blue triangles), and C_6_H_5_^+^ (orange stars). The C_7_H_7_^+^ and C_6_H_5_^+^ initially rise and then plateau at long reaction times, indicating they are terminal products of this proton transfer reaction. Conversely, C_7_H_9_^+^ displays an initial rise followed by a slow decay, indicating further reactions with neutral toluene.

**Figure 3:**
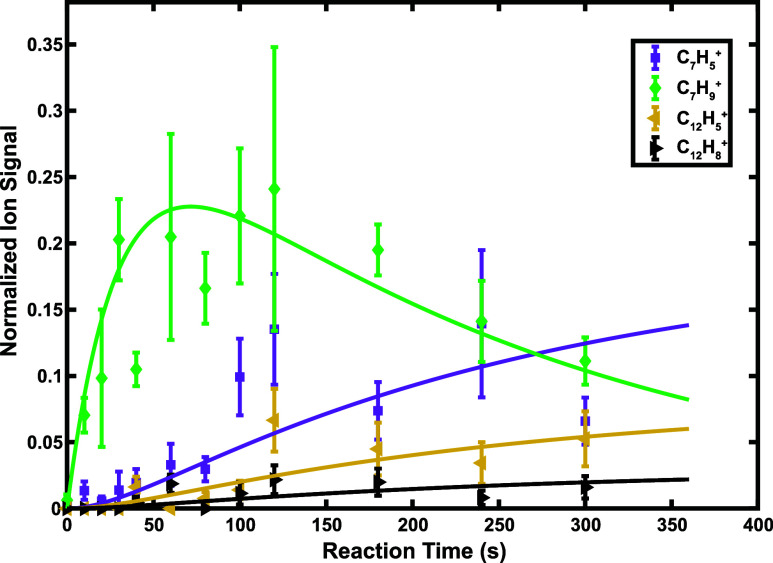
Kinetic reaction curves for the observed secondary products for reaction between C_7_H_9_^+^ + C_7_H_8_. Experimental data points were fit using a pseudo-first order rate law (curves). Each data point corresponds to seven measurements that were normalized to the fitted initial number of N_2_H^+^ ions and obtained together with the primary products. Three products are produced in this reaction, C_7_H_5_^+^ (purple squares), C_12_H_5_^+^ (gold triangles), and C_12_H_8_^+^ (black triangles). All secondary products were observed to rise continuously, indicating that the reaction time has not been extended long enough to identify either a turn over or plateau. These higher order products may continue to react, but we are unable to adequately probe their reactivity at this time because of sympathetic cooling inefficiencies with ions with *m*/*z* ≥ 115 that decreases the detection efficiency of these heavy ions.

### Secondary Products

Secondary reactions between C_7_H_9_^+^ and C_7_H_8_ were measured simultaneously with the primary proton transfer reactions, shown in [Fig fig2], but these secondary reactions have been plotted separately in Figure 3 for clarity. The assignment of these reactions as secondary is due to the delayed growth of their products as a function of time. From these secondary reactions, we observed three unique products with *m*/*z* = 89, 149, and 152. Each mass was again assigned as a pure hydrocarbon ion, with *m*/*z* = 89 assigned as C_7_H_5_^+^, *m*/*z* = 149 assigned as C_12_H_5_^+^, and *m*/*z* = 152 assigned as C_12_H_8_^+^. The observed secondary reactions are summarized in eqs 4, 5, and 6 for clarity. Note that the neutral coproducts are not directly measured in these experiments and the equations provided are examples of possible combinations, with a range of smaller neutral fragments being energetically possible as well. Further reactions were not observed within the probed reaction time.

4 $$C_{7}H_{9}^{+}+C_{7}H_{8}\to C_{7}H_{5}^{+}+C_{7}H_{12}$$

5 $$C_{7}H_{9}^{+}+C_{7}H_{8}\to C_{12}H_{5}^{+}+C_{2}H_{6}+3H_{2}$$

6 $$C_{7}H_{9}^{+}+C_{7}H_{8}\to C_{12}H_{8}^{+}+C_{2}H_{6}+H_{2}+H$$

### Reaction Rate Constants

Reaction rate constants were obtained by globally fitting all reaction data using a set of pseudo-first-order rate equations. The following set of rate equations is used to describe the primary products

7 $$\frac{dN_{C_{7}H_{7}^{+}}}{dt}=k_{1}N_{N_{2}H^{+}}$$

8 $$\frac{dN_{C_{6}H_{5}^{+}}}{dt}=k_{2}N_{N_{2}H^{+}}$$

9 $$\frac{dN_{C_{7}H_{9}^{+}}}{dt}=k_{3}N_{N_{2}H^{+}}-\left(k_{4}+k_{5}+k_{6}\right)N_{C_{7}H_{9}^{+}}$$

where *N*_N_2_H^+^_, *N*_C_6_H_5_^+^_, *N*_C_7_H_7_^+^_, and *N*_C_7_H_9_^+^_ correspond to the number of N_2_H^+^, C_6_H_5_^+^, C_7_H_7_^+^, and C_7_H_9_^+^ ions, respectively, measured via TOF-MS. The rate constants *k*_1_, *k*_2_, *k*_3_ correspond to the formation of C_6_H_5_^+^, C_7_H_7_^+^, and C_7_H_9_^+^, respectively. We observe that, of these products, only C_7_H_9_^+^ will react with neutral toluene, which results in a set of new products. The sequential reaction of C_7_H_9_^+^ with toluene is modeled using the following set of rate equations

10 $$\frac{dN_{C_{7}H_{5}^{+}}}{dt}=k_{4}N_{C_{7}H_{9}^{+}}$$

11 $$\frac{dN_{C_{12}H_{5}^{+}}}{dt}=k_{5}N_{C_{7}H_{9}^{+}}$$

12 $$\frac{dN_{C_{12}H_{8}^{+}}}{dt}=k_{6}N_{C_{7}H_{9}^{+}}$$

where *N*_C_7_H_5_^+^_, *N*_C_12_H_5_^+^_, and *N*_C_12_H_8_^+^_ are the number of C_7_H_5_^+^, C_12_H_5_^+^, C_12_H_8_^+^ ions produced from the reaction, respectively. The rate constants *k*_4_, *k*_5_, and *k*_6_ correspond to the formation of these secondary products, respectively. The concentration of neutral toluene is held constant during the entire reaction time at a partial pressure of 1.2 × 10^–9^ Torr. This ensures that all reactions proceed under a pseudo-first-order rate law and single-collision conditions.

The fitted rate constants for the primary and secondary reaction channels are summed and compared against predicted rate constants from Average Dipole Orientation (ADO) theory.^37^ We compare the results from ADO theory to our observed rate constants for: the initial proton transfer reactions (*k*_1_ + *k*_2_ + *k*_3_), and the secondary reactions with toluene (*k*_4_ + *k*_5_ + *k*_6_), tabulated in Table 1.

**Table 1:** Comparison Between the Observed Reaction Rate Constants and the Predicted Rate Constants from ADO Theory^a^

reaction	observed	ADO
N_2_H^+^ + C_7_H_8_	8.9 ± 0.6 × 10^–10^	1.81 × 10^–9^
C_7_H_9_^+^ + C_7_H_8_	1.0 ± 0.1 × 10^–10^	1.23 × 10^–9^

a All rate constants are reported with units of *cm* ^3^ *s* ^–1^. The neutral reactant concentration was measured using a Bayard-Alpert ionization gauge, which has reduced accuracy at pressures below 10^–8^ Torr.^34^ As a result, the uncertainties provided represent statistical uncertainties in the measurements and do not reflect the accuracy of the ionization gauge. Additional uncertainty is present for the secondary reactions as the detection efficiency of the products with *m/z* ≥ 115 amu is reduced.

The reaction rate constants for each individual observed reaction channel are tabulated in Table 2. Each rate constant is extracted from global fits to the data shown in Figures 2 and 3.

**Table 2:** Observed reaction rate constants for all observed reactions^a^

reaction	observed
N_2_H^+^ + C_7_H_8_ → C_7_H_9_^+^ + N_2_	2.7 ± 0.3 × 10^–10^
N_2_H^+^ + C_7_H_8_ →C_7_H_7_^+^ + H_2_ + N_2_	5.7 ± 0.6 × 10^–10^
N_2_H^+^ + C_7_H_8_ →C_6_H_5_^+^ + CH_4_ + N_2_	5 ± 1 × 10^–11^
C_7_H_9_^+^ + C_7_H_8_ →C_7_H_5_^+^ + C_7_H_12_	7 ± 1 × 10^–11^
C_7_H_9_^+^ + C_7_H_8_ →C_12_H_5_^+^ + C_2_H_6_ + 3H_2_	3 ± 1 × 10^–11^
C_7_H_9_^+^ + C_7_H_8_ →C_12_H_8_^+^ + C_2_H_6_+ H_2_ + H	1 ± 1 × 10^–11^

a All units are in *cm* ^3^ *s* ^–1^. The rate constants are determined from fitting the rate equations to all data with errors corresponding to a 68% confidence interval. The neutral reactant concentration was measured using a Bayard-Alpert ionization gauge, which has reduced accuracy at pressures below 10^–8^ Torr.^34^ As a result, the uncertainties provided represent statistical uncertainties in the measurements and do not reflect the accuracy of the ionization gauge. Additional uncertainty is present for the secondary reactions as the detection efficiency of the products with *m/z* ≥ 115 amu is reduced. The neutral co-products listed in each equation are examples of potential products and are not directly measured. A range of smaller neutral fragments are possible, especially for the secondary reactions.

## Discussion

### Primary Products

This reaction sequence begins with the formation of a reaction complex with the molecular formula C_7_H_9_^+^, from the protonation of toluene. This covalently bound complex is formed with a considerable amount of internal energy, with an ill-defined structure. In our experiment, the internal energy of this complex can be quenched only through two mechanisms, bimolecular dissociation or radiative decay. From the excited reaction complex, we observe primary products via both mechanisms −C_6_H_5_^+^ and C_7_H_7_^+^ via bimolecular dissociation and the C_7_H_9_^+^ molecule through radiative decay.

#### C_6_H_5_^+^

We observe C_6_H_5_^+^ as the least abundant of all primary products. Production of C_6_H_5_^+^ is energetically allowed (0.46 eV exothermic) with the use of a relatively strong proton donor such as N_2_H^+^, however it is not always observed depending on the experimental conditions due to the other channels being much more exothermic.^32^ Previous studies exploring the dissociation of toluene radical cations and toluenium ions have universally assigned the structure of the resulting C_6_H_5_^+^ to be the phenylium structure.^25^ Not only is phenylium the global minimum, but it is also the only energetically allowed structure for this reaction when N_2_H^+^ is used as the protonator.^38^ Isomerization to higher energy isomers of C_6_H_5_^+^ is not feasible in this case due to the presence of substantial barriers that cannot be overcome under our experimental conditions. This assignment agrees well with previous literature based on mass spectrometry and collision induced dissociation experiments.^25,39^ We have calculated a potential energy surface for this reaction at the ωB97X-D/aug-cc-pVTZ level of theory, shown in Figure 4. Based on our calculations, we found a barrierless pathway involves direct protonation at the *ipso* position of toluene, producing I 1, an *i*-toluenium-like complex and N_2_. Following the reaction between the two results in a hydrogen atom transfer from the hexagonal ring to the methyl group. This complex readily dissociates, producing phenylium and methane.

**Figure 4:**
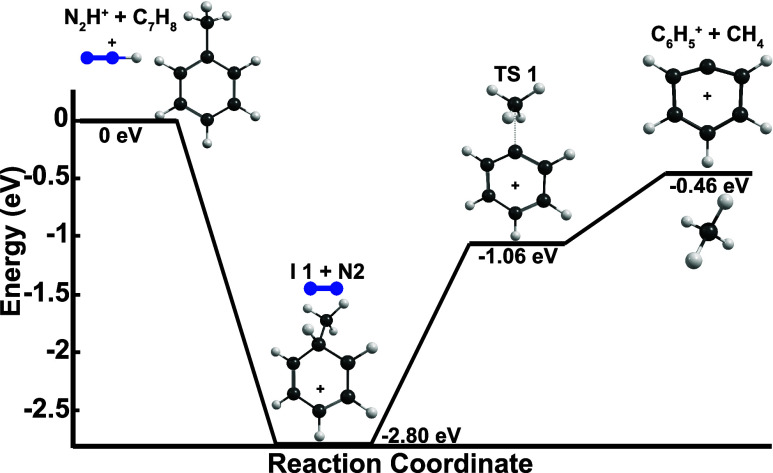
Potential energy surface for the formation of C_6_H_5_^+^ from the dissociative protonation of toluene. Protonation of neutral toluene leads to the production of I 1, an *i*-toluenium-like complex, which transfers a hydrogen atom from the ring to the methyl group through the transition state (TS 1). This complex readily dissociates to produce phenylium and methane. All calculations are performed at the ωB97X-D/aug-cc-pVTZ level of theory.

We have now produced phenylium through two very different reactions in the environment of a Coulomb crystal and found it to be highly stable in both cases.^40^ Previously, we explored the bottom-up formation of phenylium via sequential ion molecule reactions starting with C_2_H_3_^+^ reacting with C_2_H_2_, which terminated at an unreactive C_6_H_5_^+^.^40^ We tentatively assigned the structure of this C_6_H_5_^+^ as the phenylium isomer, due to stability arguments. In a Coulomb crystal, vibrational energy cannot be collisionally quenched, as the long-range Coulomb interaction between the ions is only able to quench translational energy.^41^ This means that vibrational energy can be removed only through either radiative decay or fragmentation. In our previous bottom-up study, phenylium was produced through a radiative association reaction with significant exothermicity. This means for the product to have survived in the Coulomb crystal, the reaction complex must have been able to efficiently radiate excess energy, otherwise dissociation would have readily occurred. As phenylium is Hückel aromatic, there is an inherent stability to this isomer over all others that makes it resistant to such dissociation and enables efficient radiative processes. For the current study, phenylium is the only energetically accessible product. In contrast to our previous bottom-up formation pathway,^40^ phenylium is generated here via a top-down dissociation process. This complementary formation route strengthens our assessment of phenylium’s intrinsic reactivity by enabling comparison across distinct preparation mechanisms.

Regardless of how it was formed, we have found that phenylium is highly stable and resistant to chemical reactions with several stable neutral molecules under astrophysically relevant conditions. These findings support our original claim that phenylium should be able to accumulate in the ISM to large enough abundances that enable astronomical observation.^40^ The dissociation channel resulting from protonation of toluene into C_6_H_5_^+^ also demonstrates that top-down mechanisms are able to produce monocyclic aromatic cations efficiently and can potentially be as efficient as the bottom-up equivalent.

#### C_7_H_7_^+^

The most abundant product resulting from protonation of toluene by N_2_H^+^ is C_7_H_7_^+^, which originates from a dissociative product channel distinct from the one that produces phenylium. This fragmentation channel is considered highly exothermic, and has been observed in a variety of studies using various ionization sources.^25,31,42–44^ The structure of C_7_H_7_^+^ produced in these reactions has been the subject of numerous studies in the gas-phase due to the possibility of forming two highly stable isomers, tropylium and benzylium.^45,46^ In most cases, the global minimum isomer, tropylium, was produced exclusively regardless of method due to its high stability relative to other isomers.^47^ It was later shown that benzylium could be readily produced under kinetically controlled conditions.^27,46^ These observations motivated a series of studies to develop methods to identify specific isomers of C_7_H_7_^+^. Over time, the reaction between C_7_H_7_^+^ and toluene was found to be the most effective method, as it resulted in a binary response. If the tropylium isomer was produced, it would not react with toluene. If the benzylium isomer was produced, it would react to form C_8_H_9_^+^.^47^

With our experimental conditions, there are eight energetically accessible isomers of C_7_H_7_^+^.^48^ Of these, all are expected to react readily with toluene except for the tropylium cation. We confirm that tropylium is the sole isomer produced in our experiment. As shown by the blue curve in Figure 2, the C_7_H_7_^+^ signal remains constant over time indicating no reaction is taking place. Knowing that we produce only tropylium, we compute a potential energy surface and find a barrierless pathway to produce tropylium from this dissociative proton transfer to toluene, shown in Figure 5. This barrierless pathway begins with the formation of I 1, an *o*-toluenium-like complex, which then forces a ring expansion to form I 2. The complex continues to rearrange around the heptagonal ring structure until H_2_ elimination occurs, shown as TS 4, finally resulting in the tropylium structure.

**Figure 5:**
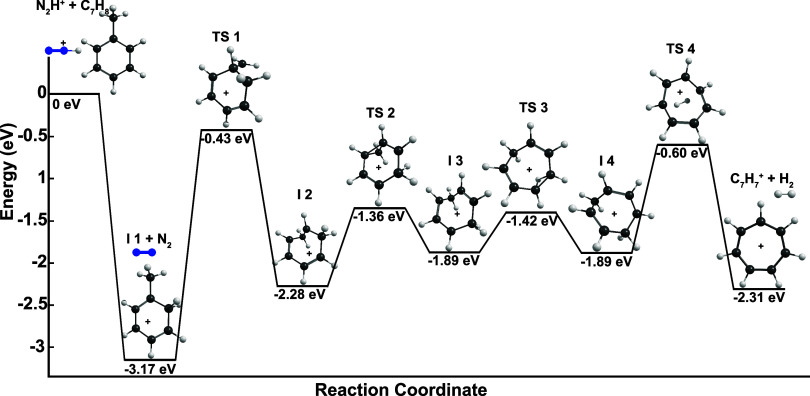
Potential energy surface for the formation of C_7_H_7_^+^ from the dissociative protonation of toluene. Protonation of neutral toluene leads to production of I 1, an *o*-toluenium-like complex, which leads to opening of the hexagonal carbon framework into a heptagonal ring, followed by H_2_ elimination. All calculations are performed at the ωB97X-D/aug-cc-pVTZ level of theory.

#### C_7_H_9_^+^

We observe an initial growth of C_7_H_9_^+^ followed by a slow decay after 100 s of reaction time, indicating a reaction occurring between C_7_H_9_^+^ and C_7_H_8_(As shown in Figure 2). Unlike phenylium and tropylium, none of the isomers of C_7_H_9_^+^ are π-aromatic, making C_7_H_9_^+^ inherently less stable compared to the other observed primary products. This instability is further demonstrated by C_7_H_9_^+^ being the only primary product that reacts with neutral toluene in this study.

Formation of C_7_H_9_^+^ is the result of an associative proton transfer reaction, in which C_7_H_9_^+^ can appear as several different isomeric forms based on energetics.^25^ However, previous work by Mormann et al. have demonstrated that direct protonation to toluene results in only toluenium-type isomers and does not result in other ring expanded or ring contracted structures.^26^ Toluenium ions have been studied with a variety of formation methods by mass spectrometry, with all previous studies finding a mixture of tautomers formed, and the ratio of tautomers varying between formation methods.^25^ Toluenium ions have been produced through chemical reactions, supersonic expansion, and photodissociation experiments and then characterized in the infrared by IRMPD and IRPD, which found that there were a mixture of tautomers produced regardless of production method.^27,49–51^

In our experiments, it is unlikely that a mixture of toluenium tautomers is present, given the mechanism of energy removal in our Coulomb crystal system. Although tautomerization of toluenium is barrierless following protonation of toluene (see Figure 6), the excess energy deposited during protonation will be removed through radiative relaxation before the ion undergoes a subsequent collision with a neutral reactant. Because the collision rate in our trap is extremely low (∼ 1 Hz), radiative decay is the dominant energy dissipation pathway.

**Figure 6:**
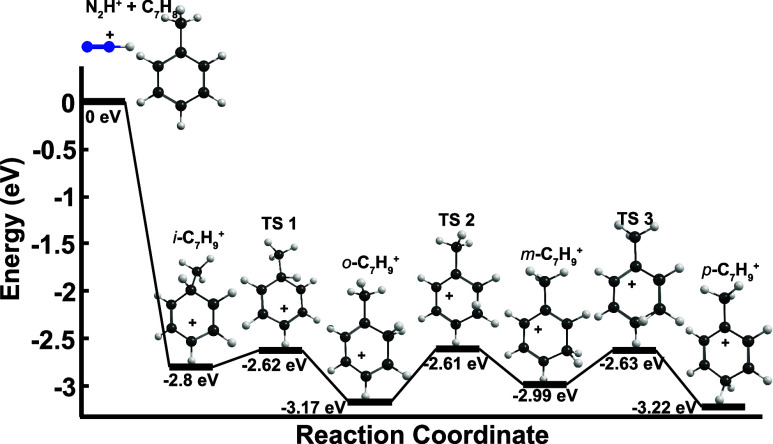
Potential energy surface for the protonation of toluene leading to four potential tautomers of the toluenium cation (C_7_H_9_^+^). The calculated transition states demonstrate negligible barriers for the proton ring walk to occur when generating toluenium from protonation by N_2_H^+^. All structures and transition states are computed at the ωB97X-D/aug-cc-pVTZ level of theory.

Radiative vibrational cooling is therefore expected to stabilize the lowest-energy tautomer. Both theoretical calculations and condensed phase studies identify *p*-toluenium as the global minimum.^28–30^ While we cannot definitively assign the structure of the toluenium ions formed in our experiment, the dominance of radiative decay strongly suggests that the lowest-energy tautomer is preferentially populated. Because tautomerization is barrierless, any initially formed structure can interconvert prior to full vibrational relaxation, ultimately favoring the most stable form.

At the ωB97X-D/aug-cc-pVTZ level of theory, *p*- and *o*-toluenium differ in energy by only 0.06 eV, effectively rendering them degenerate within expected theoretical uncertainty. As a result, we cannot conclusively distinguish between these two tautomers based solely on calculated energetics. A more definitive structural assignment will require improved characterization of the relative energetics and reactivity of individual toluenium tautomers.

### Secondary Products

#### C_7_H_5_^+^

Although the reaction between C_7_H_9_^+^ and C_7_H_8_ was anticipated to form larger, potentially polycyclic hydrocarbons as happens with neutral–radical chemistry, we find that a substantial fraction of the reaction instead yields a smaller ion, C_7_H_5_^+^. There are very few reaction studies that have produced C_7_H_5_^+^ in the gas-phase. However, all previous studies share a similar reaction mechanism, carbon atom insertion to neutral benzene.^52–54^ This has led to the proposal that the C_7_H_5_^+^ would have heptagonal ring structure, similar to the tropylium ion.^53^ Very little theoretical work has been carried out on possible structures of C_7_H_5_^+^. There are several potential isomers that could be formed in this experiment based on prior calculations.^54^ A detailed study has been performed on the fulvenallyl radical structure, which mirrors the global minimum of neutral C_7_H_5_.^55^ However, calculations at the CCSD­(T)/CBS//CCSD/aug-cc-pVTZ level of theory show a different structure as the global minimum for the cation, which has never been observed spectroscopically.^54^ At this time, the exact structure of C_7_H_5_^+^ produced in this study cannot be definitively determined, which also prevents any mechanistic interpretation of its formation.

#### C_12_H_5_^+^ and C_12_H_8_^+^

In addition to a smaller product ion being produced from the reaction between C_7_H_9_^+^ and C_7_H_8_, we also observe heavier ions, C_12_H_5_^+^ and C_12_H_8_^+^, which indicates that potential polycyclic growth can occur through these reactions. Unfortunately, we are unable to confirm that polycyclic structures are formed because we do not have a means of characterizing the structures of these products. This is largely because these ions have little to no data on their structures available in the literature, both experimentally and theoretically. To the best of our knowledge C_12_H_5_^+^ has not been characterized experimentally or theoretically. C_12_H_5_^+^ has been observed previously as a product resulting from a reaction between C_8_H_5_^+^ + C_4_H_2_, but potential structures were not characterized.^56^ Based on the size of the molecule alone, we expect there are many possible isomers, which we are unable to distinguish at this time.

Several isomers of C_12_H_8_^+^ have been observed in the gas-phase using various spectroscopic techniques. The acenaphthylene radical cation has been studied experimentally with IRMPD and IRPD and found to have a dense and complicated spectrum.^57^ Both acenaphthylene and biphenylene have been studied by photoelectron and matrix isolation spectroscopies.^58–60^ Isomers of ethynylnaphthalene have been characterized by IRPD and found to be prone to isomerization at high energy.^61,62^ Isomers of C_12_H_8_^+^ have also been explored computationally, with the acenaphthylene isomer being identified as a target for astronomical observation due to calculations predicting it to have the highest photostability, which would allow it to survive in high flux regions of the ISM.^63^ Experiments in cryogenic ion storage rings have shown that the dissociation and radiative stabilization rates of both acenaphthylene and biphenylene cations are very similar and interconversion between the isomers appears to be slower than unimolecular dissociation.^64^ Even with all this information, we are unable to determine the isomer of C_12_H_8_^+^ formed in our experiments, as none of the previous studies have produced C_12_H_8_^+^ through bottom-up style reactions, where formation of multiple species is energetically possible.

In the context of chemistry in the ISM, relatively little is known about the top-down or bottom-up formation of these ions or potential isomerization pathways between different structures. Two cyano-acenaphthylene isomers were recently observed in the ISM and it has been proposed that ion–molecule reactions involving C_12_H_8_ could be responsible for the formation of c-C_11_H_8_, which was also recently been detected in TMC-1.^65,66^ However, given the lack of experimental and theoretical support, we are unable to justify the formation of this structure over other potential isomers that have been observed in other experiments.

We are also unable to measure an accurate branching ratio for the secondary reactions due to low detection efficiency of heavy masses in our instrument. In our experiment, product ions are sympathetically cooled via Coulomb interactions with laser-cooled Ca^+^, substantially reducing their translational motion. However, the efficiency of sympathetic cooling decreases as the mass difference between Ca^+^ and the cotrapped ion increases. As a result, heavier ions are cooled less effectively and occupy larger radial positions in the trap. During extraction into the TOF-MS, particularly heavy ions (≥115 amu) are, therefore, more likely to strike the rod electrodes and fail to reach the detector. Because both C_12_H_5_^+^ and C_12_H_8_^+^ lie well above this mass threshold, we expect reduced detection efficiency for these species. We observed a similar effect when detecting Xe^+^ stored in our trap.^67^

Regardless of the precise structures of C_12_H_5_^+^ and C_12_H_8_^+^, the formation of polycyclic, pure hydrocarbon ions from ion–molecule reactions in the gas-phase is both interesting and important to the formation of PAHs in the ISM. This reaction indicates that there are unexplored pathways that can result in the formation of polycyclic ions in the gas-phase, which should be relevant to the formation mechanisms of PAHs in the ISM. A deeper understanding of both potential structures of these ions, as well as their reactivity will be necessary to identify their role in the formation of PAHs in space.

## Conclusion

We have explored ion–molecule reactions involving toluene and toluenium ions to probe potential formation mechanisms of PAHs in the ISM. For the protonation reaction, we find three unique products: C_7_H_9_^+^, C_7_H_7_^+^, and C_6_H_5_^+^. All of these products have been observed previously in various ion–molecule reaction experiments. We also explored these products reactions with neutral toluene. We found that neither C_7_H_7_^+^ nor C_6_H_5_^+^ reacted with neutral toluene, allowing us to assign the structures as tropylium and phenylium, respectively. The toluenium ion, on the other hand, did react with neutral toluene to form three unique products. In addition to the formation of C_7_H_5_^+^, two larger hydrocarbon ions were produced, C_12_H_5_^+^ and C_12_H_8_^+^, which indicated a potential formation of polycyclic molecules. This finding points to the presence of a new class of hydrocarbon ion–molecule reactions that extend beyond formation of benzene. Although toluene has yet to be detected in the ISM at this time, this study highlights the possibility that alkylbenzenium ions could play a role in the formation of PAHs throughout the universe.

## Supplementary Material



## Notes

Example mass spectrum obtained at t = 20 seconds (PDF)
