# Supplementary material for: Formation of Monocyclic and Polycyclic Hydrocarbons from Sequential Toluene Reactions in Coulomb Crystals
Source: J Phys Chem A. 2026 May 19;130(22):4162–70. doi: 10.1021/acs.jpca.6c01167 (PMC13244555; doi:10.1021/acs.jpca.6c01167)
Supplement: Supplementary file 1 [file jp6c01167_si_001.pdf]

Formation of monocyclic and polycyclic hydrocarbons from sequential toluene reactions in Coulomb crystals

G. S. Kocheril[1,2], C. Zagorec-Marks[1,2], S. H. Allison[1,3], and H. J. Lewandowski*[1,2]

[1]JILA, National Institute of Standards and Technology, and the University of Colorado, Boulder, Colorado 80309, United States of America
[2]Department of Physics, University of Colorado, Boulder, Colorado 80309, United States of America
[3]Department of Chemistry, University of Colorado, Boulder, Colorado 80309, United States of America

*Email: lewandoh@colorado.edu

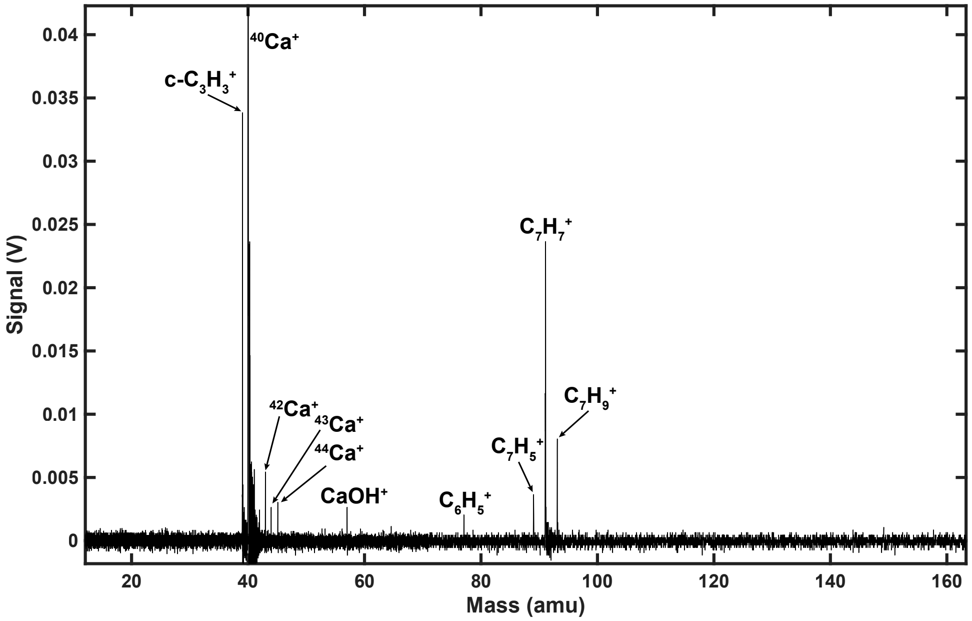

Figure 1: Example mass spectrum obtained at t=20 seconds. The $^{40}Ca^{+}$ peak is larger than the vertical axis to be able to show the small signals from the other ion peaks. In addition to the product ion peaks, signal from various Ca^{+} isotopes are observed. The peak at m/z = 57 is assigned as $CaOH^{+}$, which is the result of the Ca^{+} Coulomb crystal reacting with trace water present in the vacuum chamber. The peak at m/z=39 is assigned as c-$C_3H_3{}^{+}$. This peak is assigned to be the result of contamination arising from the 95% toluene solution used in this study. This signal appears only when toluene is admitted into the vacuum chamber, regardless of whether the Coulomb crystal is present or not. The specific formation mechanism was not explored in this study, but this reaction does not interfere with the main series of reactions.
